# Zinc in Gut-Brain Interaction in Autism and Neurological Disorders

**DOI:** 10.1155/2015/972791

**Published:** 2015-03-23

**Authors:** Guillermo Vela, Peter Stark, Michael Socha, Ann Katrin Sauer, Simone Hagmeyer, Andreas M. Grabrucker

**Affiliations:** ^1^Zinpro Corporation, Eden Prairie, MN 55344, USA; ^2^Autismo ABP, 64639 Monterrey, NL, Mexico; ^3^WG Molecular Analysis of Synaptopathies, Neurology Department, Neurocenter of Ulm University, 89081 Ulm, Germany; ^4^Institute for Anatomy and Cell Biology, Ulm University, 89081 Ulm, Germany

## Abstract

A growing amount of research indicates that abnormalities in the gastrointestinal (GI) system during development might be a common factor in multiple neurological disorders and might be responsible for some of the shared comorbidities seen among these diseases. For example, many patients with Autism Spectrum Disorder (ASD) have symptoms associated with GI disorders. Maternal zinc status may be an important factor given the multifaceted effect of zinc on gut development and morphology in the offspring. Zinc status influences and is influenced by multiple factors and an interdependence of prenatal and early life stress, immune system abnormalities, impaired GI functions, and zinc deficiency can be hypothesized. In line with this, systemic inflammatory events and prenatal stress have been reported to increase the risk for ASD. Thus, here, we will review the current literature on the role of zinc in gut formation, a possible link between gut and brain development in ASD and other neurological disorders with shared comorbidities, and tie in possible effects on the immune system. Based on these data, we present a novel model outlining how alterations in the maternal zinc status might pathologically impact the offspring leading to impairments in brain functions later in life.

## 1. Introduction

Research from the last decades clearly shows that zinc has a vital role in neonatal development. Zinc is an essential trace element in humans and animals and is involved in countless metabolic and signaling pathways within the body. However, a particular role of zinc in the immune system and brain has been reported [1]. Zinc is one of the most prevalent metal ions in the brain and participates in the regulation of neurogenesis, neuronal migration, and differentiation, thereby shaping cognitive development and maintaining healthy brain function. Zinc deficiency during pregnancy results in specific impairments in the offspring, which have been observed in animal models but might also be present in humans [2]. Intriguingly, among individuals with Autism Spectrum Disorders (ASD), the incidence rate of zinc deficiency has been reported to be significantly increased compared to age matched healthy control subjects [3]. The occurrence of zinc deficiencies in ASD is particularly pronounced in very young age [4, 5], where a rate of almost 50% was reported in the age group of 0–3 years [5]. These low levels of zinc often occur along with copper overload and the Cu/Zn ratio was reported to correlate with the severity of symptoms associated with autism [6–8]. This early occurrence of zinc deficiency with decline later in life and the manifestation of some of the core features of ASD, such as impaired social behavior and language and communication problems in prenatal zinc deficient mice [9], have recently put maternal zinc status in the focus as a possible environmental factor in the etiology of ASD. Thus, maintaining adequate zinc status during pregnancy might be a promising approach to prevent cognitive and neurobehavioral deficits later in life. However, meeting the zinc requirement of the mother can be challenging.

Two major pools of zinc can be found within the body: a slowly zinc exchanging pool that contains about 90% of the body's zinc and a pool that rapidly exchanges zinc with the plasma. The latter, which contains the other 10% of zinc, is the one that is especially reactive to the amount of absorbed zinc and is the first to be depleted under conditions of zinc deficiency. Plasma zinc is also the source of the embryo's zinc supply. In order to maintain proper zinc levels during pregnancy, both endogenous losses and the increased demand resulting, for example, from synthesis of novel tissue must be covered by absorption of zinc from dietary sources. Thus, while the metabolic zinc requirement of 2.5 mg/d for an adult woman is generally met when consuming daily 10 to 15 mg zinc, due to the additional need for zinc during pregnancy, an additional 5–10 mg zinc per day must be consumed to meet the increasing demand of 0.08, 0.24, 0.53, and 0.73 mg of metabolic zinc per day for the four quarters of pregnancy [10]. Similarly, during lactation, the metabolic daily requirement increases by another 2.5 mg per day. Meeting these requirements is challenged by several factors. First, it is not uncommon for women of childbearing age to consume low zinc diets. Second, zinc status of women may be compromised due to increased intake of dietary constituents that reduce the availability of zinc.

Impact of low zinc status of the mother can be magnified depending on time and severity of the deficiency, ranging from teratogenic effects with severe deficiency to functional impairments acting, for example, on brain development with mild deficiency. In particular, teratogenic effects have been reported in rodent models [11, 12] as well as in humans, where women with Acrodermatitis enteropathica, a genetic disorder resulting in impaired zinc absorption, show a high incidence of birth defects [13]. In general, although the brain seems most vulnerable, all organ systems are affected by systemic zinc deficiency in times of active proliferation and differentiation. Thus, although mild zinc deficiency does not lead to gross morphological malformations in the offspring, the reported behavioral impairments might result from a combination of alterations in brain development and other organ systems. This novel vista on the role of zinc deficiency in ASD broadens the focus from the action of zinc within the brain to other organs such as the GI system.

Proper zinc status is necessary for healthy gut development and both pre- and perinatal zinc deficiency might affect the neonate and potentially trigger downstream events that contribute to pathological processes [14]. These processes may, among others, include inflammation due to increased intestinal epithelium permeability and immune system abnormalities including the generation of autoantibodies. Another consequence of impaired or delayed gut development will be lowered trace metal absorbance, which might contribute to the slow normalization of biometals in children with ASD after birth [5]. GI discomfort, changes in gut microbiome, food aversion, and an increased intestinal permeability have been shown to correlate with the severity of behavioral symptoms in individuals with ASD [15–21].

Given that inflammatory cytokines and other immune signaling molecules originating from the GI tract interact with the hypothalamic-pituitary-adrenal gland (HPA) stress axis, prenatal stress itself can be integrated in this pathomechanism, targeting the same structures [22]. Thus, some of the major environmental risk factors for the development of ASD are linked in this model.

Taken together, maternal zinc deficiency might impair the gut development of the offspring and thereby increase the risk for GI problems, inflammatory events, abnormal immune signaling, trace metal imbalances, and ultimately altered brain function. Data supporting this hypothesis will be discussed further in more detail.

## 2. Zinc and Gut Formation

**Figure 1:**
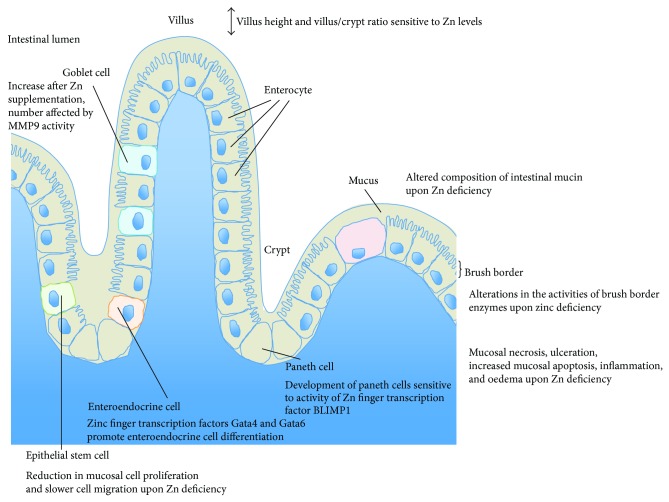
Influence of zinc levels on gut formation. Zinc levels mediate villus height and villus/crypt ratio in the jejunum. Zinc deficiency results in a shortening and narrowing of the villi and thus a reduction in absorptive surface. This may be mediated by a reduction in mucosal cell proliferation and slower cell migration, as well as an increase in the number of apoptotic cells in villi and crypts. The zinc finger transcription factors Gata4 and Gata6 are involved in intestinal epithelial cell differentiation and promote enteroendocrine cell differentiation. Moreover, the number of goblet cells increases after zinc supplementation and is dependent on the activity of the zinc binding matrix metalloproteinase-9 (MMP-9). Goblet cells secrete mucins and an altered composition of intestinal mucin was reported in zinc deficient animals. Additionally, several alterations in the activities of brush border enzymes result from zinc deficiency. The development of paneth cells is accelerated by the zinc dependent transcription repressor BLIMP1. Furthermore, zinc deficiency is accompanied with mucosal necrosis and ulceration, inflammation, and oedema.

A well-orchestrated sequence of highly specialized processes is required for the development of the intestine from the embryonic gut tube to a complex organ responsible for food digestion and absorption of essential nutrients. Particularly the sophisticated intestinal epithelium is strongly dependent on a proper development in order to fulfill its widespread functions ranging from defense of antigens to absorption of important nutrients. These processes are dependent on the correct sequence of cell proliferation, differentiation, and apoptosis. Given that these processes require a plethora of zinc dependent enzymes, it is quite obvious that zinc deficiency especially during the embryonic development of the gut might lead to alterations in intestinal morphology and cell composition resulting in possible functional alterations (Figure 1). Unfortunately, only very limited data is available on the precise effects of prenatal zinc deficiency during fetal development and differentiation of the small intestine.

Intriguingly, researchers found that feeding sows an additional 250 ppm zinc from zinc amino acid complex during the last trimester of pregnancy resulted in improved intestinal development of pigs. The offspring of sows fed the additional zinc had increased villous height and villus/crypt ratio in the jejunum and higher goblet cell counts in the ileum [23]. Furthermore, intestinal defenses of these pigs against pathogens appeared to have been improved as indicated by an increased number of intraepithelial lymphocytes in the duodenum and ileum.

However, most of the available data on the role of zinc in gut development originate from induced zinc deficiency in immature and mature animals. Several studies have shown fatal consequences of acute and chronic zinc deficiency on the structure and function of the small intestinal epithelium. For example, zinc is crucial for the maintenance of the small mucosal integrity [24–26] and zinc deficiency accompanied with mucosal necrosis and ulceration as well as increased mucosal apoptosis, inflammation, oedema, and structural alterations of villi. Thus, it is not surprising that zinc supplementation has been shown to have beneficial effects on mucosal integrity in many pathophysiological and inflammatory conditions of the small intestine [26, 27]. Further, individuals with Acrodermatitis enteropathica who suffer from severe zinc deficiency showed villus atrophy and gut necrosis [28].

Zinc deficiency also results in morphological and functional changes of the intestinal epithelium. When zinc deficient diet is fed to immature male rats for 28 days, a significant reduction of small intestinal length and further morphological changes in the jejunum, including shortening and narrowing of the villi, reduction in absorptive surface, and an increased number of villi per unit area of serosa, were reported that could be restored by zinc supplementation [29, 30]. Further, a reduction in mucosal cell proliferation and slower cell migration were shown [29]. Moreover, ultrastructural changes on a cellular level, such as appearance of membrane-bound autophagic vacuoles, pyknotic nuclei, and dilated nuclear periphery can be observed in zinc deficient rats [31]. Additionally, a study in zinc deficient rats and sheep revealed an altered composition of intestinal mucin hinting towards functional alterations in mucin-secreting goblet cells [32]. Goblet cells reside throughout the GI tract producing a protective mucus blanket. This mucin-containing mucus layer has an important role in innate host defense.

Zinc deficiency leads to a reduction in crypt cell proliferation [30]. A factor contributing to this impairment might be an increase in the number of apoptotic cells in villi and crypts, especially in the midzone of the crypts that serves as the zone of renewal of the intestinal epithelium [33, 34]. This hints towards a reduced renewal capacity of the intestinal epithelium that is required for its proper function. A positive effect of zinc supplementation on the repair capacity of the small intestine, especially the third segment, has been reported in mice [35]. These mice showed a higher intestinal epithelium cell production rate and shorter duration of mitosis compared to their control littermates [35]. Thus, besides the effects of zinc deficiency on morphology of the small intestine, the maintenance and repair capacity of the epithelium are affected.

During development, small intestine maturation is measured by indicators like increased cell proliferation and differentiation as well as an altered activity of brush border disaccharidases like lactase and sucrase [36] due to changing nutritional demands. Lactase and sucrase serve as markers of enterocyte maturity and functional capacity as well as villus height and crypt depth [36]. Several alterations in the activities of brush border enzymes have been reported to result from zinc deficiency. Chronic zinc deficiency, for example, reduces the activity of disaccharidases like sucrase, trehalase, lactase, leucine aminopeptidase, alkaline phosphatase, and maltase by 30–50% at the brush border of the small intestine [34, 36, 37]. Correct function of intestinal disaccharidases is inevitable for proper digestion of carbohydrates and absorption of saccharides. Further, the zinc dependent metalloenzyme alkaline phosphatase showed similar reduction in activity. Given that zinc is crucial for maintenance of membrane structure and function, the loss of brush border integrity due to zinc deficiency might lead to the dysfunction of these enzymes [37] and thus altered gut maturation.

Additionally, many genes regulating the differentiation into intestinal epithelium in adults as part of a self-renewal process of the epithelium by intestinal stem cells localized in the base of crypts also play a crucial role in the regionalization of the gut during the development [38]. Zinc dependent transcription factors are highly involved in the regulation of these genes and their dysfunction has severe consequences on intestinal development. For example, the transcription factors Gata4 and Gata6 are involved in the proximal-distal specification of the intestine as well as epithelial cell differentiation [38]. Gata4 seems to regulate sucrase-isomaltase and lactase transcription hinting towards a role in maturation of the enzymatic brush border composition [39, 40] and the loss of Gata4 leads to decreased absorption of cholesterol and fats [41]. Furthermore, the conditional knockout of Gata4 and Gata6 results in reduced promotion of enteroendocrine cell differentiation [42].

A further zinc dependent transcription repressor, B lymphocyte-induced maturation protein 1 (BLIMP1), is required to delay the final maturation of suckling to weaning intestinal epithelium allowing the dietary transition from mother's milk to solid diet and is therefore specifically expressed in developing and postnatal intestine [43]. BLIMP1 knockout mice are born with features resembling an adult intestine such as more serrated appearance of villi and accelerated development of paneth cells [43, 44]. During suckling period, the expression of disaccharidases, typically expressed in postweaning periods, is upregulated in BLIMP1 knockout mice whereas the expression of disaccharidases important for lactose digestion is lost [43, 44].

Additional zinc dependent transcription factors like the growth factor independent 1 (Gfi-1) and Mtgr1 are involved in secretory cell differentiation [45, 46]. Although zinc is bound within zinc finger transcription factors with high affinity and only potent zinc chelators are able to resolve zinc binding, it might be possible that, along normal protein turnover, severe zinc deficiency leads to less stable and/or functional transcription factors. Further, zinc binds with less affinity to enzymes like class I histone deacetylases that have been reported to be involved in the regulation of intestinal epithelium differentiation [47]. Additionally, activity of the zinc binding matrix metalloproteinase 9 (MMP-9) seems to influence the number of goblet cells and by that increases secretion of the mucin Muc-2 [48]. However, it has to be mentioned that despite the beneficial effects of zinc supplementation on small intestinal epithelial structure, excessive amounts of zinc can lead to damage* in vitro* [49]. Thus, the appropriate zinc status during development is crucial for a healthy functional intestine.

Taken together, zinc deficiency in animals and humans has strong effects on the intestinal epithelium structure and function (Figure 1). Due to these severe consequences it is likely that zinc deficiency during embryologic development might lead to morphological alterations resulting in functional impairment of the small intestine. These impairments might include malabsorption of essential nutrients leading to malnutrition, diarrhea, and inflammation in the immature gut.

## 3. Gut-Brain Interaction in ASD and Other Neurological Diseases

A growing amount of research indicates that at least a portion of the dysfunctions associated with ASD is related to GI problems [50]. However to date it remains unclear whether GI problems are comorbidities or a causative pathomechanism of ASD [50, 51]. It has been repeatedly reported that children with ASD frequently suffer from GI problems such as diarrhea, constipation, bloating, abdominal pain, and gastroesophageal reflux [52, 53]. GI problems (based on parents' reports) were identified in 42% of children and 12% of controls, with constipation (20%) and chronic diarrhea (19%) being the most common symptoms [17]. Furthermore, altered intestinal barrier function has been found in subjects with ASD [54] along with an increased intestinal permeability [55]. Another contributing factor to GI problems in individuals with ASD might be an abnormal composition of gut microbiota. In the GI flora of autistic children, using stool samples, lower levels of beneficial* Bifidobacter* species and higher levels of* Lactobacillus* species were found compared to controls. Other studies stated altered* Clostridium* species numbers and types in children with ASD [53–58] and differences concerning the phylum level with an increase in* Bacteroides* and a decrease in* Firmicutes* in the ASD group [59–61]. It has been stated that GI disturbances correlate with the severity of ASD. The stronger the GI symptoms are, the more likely children presented severe autistic symptoms [53].

The GI tract plays an important neurological function and therefore sometimes is referred to as the “the second brain.” Via enteric nerves and networks, the GI tract is able to affect the brain and vice versa [62, 63]. Intriguingly, in healthy human subjects, modulation of the gut microbiome was shown to have the potential to alter brain responsiveness to an emotion recognition task [64].

Besides the GI disorders often found in ASD patients, the gut-brain interaction seems to play a role in other neurological disorders as well [65]. Investigating the prevalence of depression and anxiety disorders as comorbidity in inflammatory bowel disease (IBD), individuals with Crohn's disease and ulcerative colitis, two types of IBD, are more likely to suffer from psychiatric disorders like depression and anxiety disorders in comparison to the general population [66–68]. When comparing the comorbidity of the GI disorders IBD and irritable bowel syndrome (IBS), significantly more subjects were diagnosed with depression (IBS: 61%; IBD: 16%), generalized anxiety disorder (IBS: 54%; IBD: 11%), panic disorder (IBS: 61%; IBD: 11%), and agoraphobia (IBS: 25%; IBD: 25%) [69], with a higher prevalence for a lifetime diagnosis of the aforementioned comorbidities in comparison to the general population [68, 69].

Intriguingly, along with the core features of ASD, comorbidities occur frequently in ASD patients such as seizures, depression, and anxiety disorders that have been associated with zinc deficiency before.

## 4. Zinc, the GI Tract, Stress, and the Immune System

Zinc deficiency severely affects almost all components of the immune system. Even marginal zinc deficiency leads to a seriously depressed immune system. Thus, the susceptibility to infections is increasing with a decreasing zinc status as reported from animal models and human studies. The vulnerability to infections is associated with an impaired T and B lymphocyte development and differentiation and their reduced activity [70–72] whereby T lymphocytes seemed to be more seriously affected [72] by zinc deficiency. Studies on prenatal zinc deficient animals have shown that zinc deficiency, even a marginal one, results in smaller lymphoid organs and less immunoglobulins [73]. Beneficial effects of zinc supplementation in diseases include reduced incidence and duration of acute and persistent diarrhea [74–76], reduced incidence of acute lower respiratory infections [77], and reduced duration of the common cold [78].

A role of abnormal immune system function in ASD has long been hypothesized. In postmortem brains of individuals with ASD, similar to some animal models, activation of astroglia and microglia was reported, indicating some degree of neuroinflammation [79–82]. Furthermore, a relationship between familial autoimmune disorders and anti-inflammatory/immune-modulating drug in ASD has been reported [83]. Inflammatory events however, can also be mediated by abnormalities in the GI system. Usually the organism fight against pathogens is initiated by the activation of the complement system as well as natural killer cells and polymorphonuclear leukocytes. All these defending mechanisms are depressed by zinc deficiency [84–87] resulting in a prolonged inflammation. The disruption of these processes is also associated with diarrhea or inflammatory bowel disease, both also consequences of zinc deficiency.

Inflammation in the GI tract can lead to intestinal permeability, commonly called “leaky gut.” Here, increased spaces present between cells in the small intestine may result in incompletely broken down foods and other toxins entering the blood stream, which may lead to an immune system response [16] triggering the release of antibodies. This process may result in chronic inflammation that might influence microbe proliferation in the GI tract and cause vitamin and mineral deficiencies, as well as food allergies and autoimmune diseases such as celiac disease [88]. Furthermore, a direct effect on the brain causing behavioral, cognitive, and psychiatric impairments may occur [89, 90].

Additionally, gut inflammation and zinc deficiency are also linked to physiological and psychological stress. Animal studies have shown that psychological stress decreased serum zinc levels [91]. Reduced zinc levels and psychological stress both increase the release of glucocorticoids [92, 93]. Increase levels of glucocorticoids in turn have been associated with thymic atrophy and reduced B lymphocyte numbers [92, 94, 95]. Furthermore, persistent high levels of glucocorticoids might lead to resistance of glucocorticoid receptors and in turn lead to failure of immune system downregulation. This downregulation is necessary to avoid chronic inflammatory processes.

**Figure 2:**
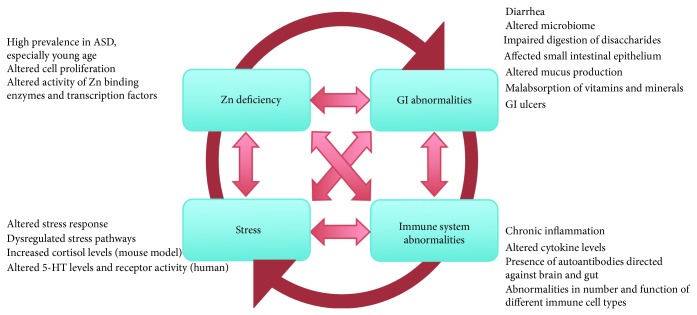
GI abnormalities, immune system dysfunction, stress, and zinc deficiency may be highly linked processes contributing to the development of ASD. Zinc deficiency mediates GI system abnormalities, severely affects many components of the immune system, and is linked to physiological and psychological stress. Although there is good reason to believe that maternal zinc deficiency might be the initial trigger, once this vicious cycle is activated in the offspring, GI abnormalities, impaired immune system, stress, and zinc deficiency can be both cause and consequence of each other and influence the development of ASD. This is in line with the often reported symptoms and comorbidities in ASD associated with problems linked to these four key features.

Taken together, GI abnormalities, immune system dysfunction, stress, and zinc deficiency are highly linked processes (Figure 2). The final result may be an altered signaling to and within the developing brain, possibly contributing to the development of ASD.

## 5. Conclusions

### 5.1. A Model for Zinc in Gut-Brain Interaction in ASD

**Figure 3:**
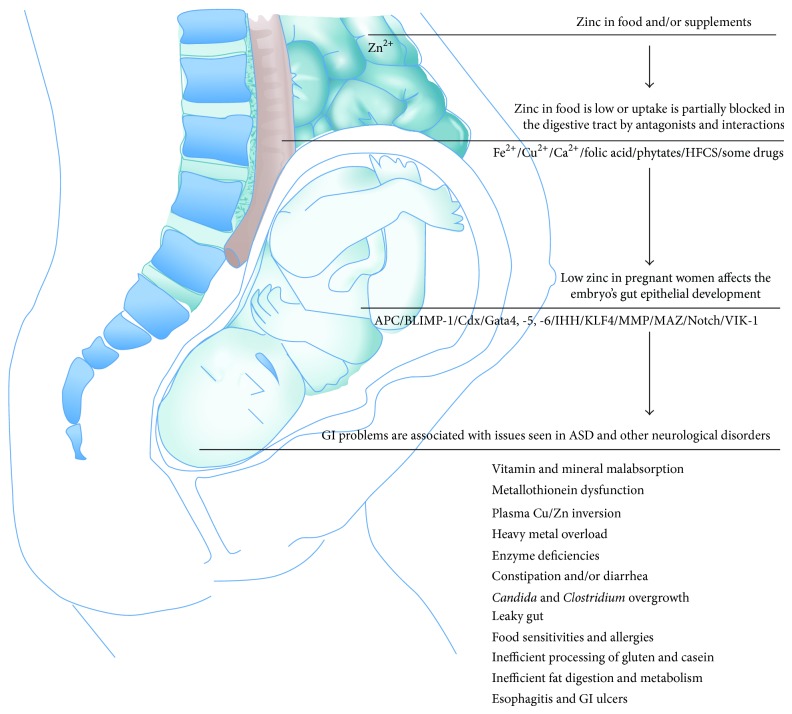
A model for Zinc in gut-brain interaction in ASD and other neurological disorders. Zinc is taken up from our dietary sources and/or supplements in the proximal small intestine. However, absorption of zinc can be decreased in response to various agents such as iron and/or calcium supplements, high copper levels, folic acid, phytate, high fructose corn syrup (HFCS), and/or several drugs. Alternatively, zinc levels may be low due to genetic variants in zinc homeostasis genes or general low availability of zinc in the diet. As a result of this, zinc deficiency of the embryo may occur. Zinc deficiency might influence embryonic and fetal development affecting the GI system through impaired function of several key proteins contributing to many of the reported GI problems associated with ASD such as metallothionein dysfunction, plasma Cu/Zn inversion, heavy metal overload,* Candida* and* Clostridium* overgrowth, constipation and/or diarrhea, leaky gut, food sensitivities and allergies, inefficient processing of gluten and casein, enzyme deficiency, vitamin and mineral malabsorption, inefficient fat digestion and metabolism, and esophagitis and GI ulcers. These GI symptoms can give rise to behavioral difficulties.

Due to the multifaceted effect of zinc on gut development and morphology, pre- and perinatal zinc deficiency might affect gut development of the neonate and potentially mitigate many of the dysfunctions shared between ASD and other neurological disorders. Based on this hypothesis, a model emerges (Figure 3) that might serve as starting point for future studies.

Zinc is taken up from our dietary sources and/or supplements in the proximal small intestine, either the distal duodenum or proximal jejunum [96, 97]. Within enterocytes, intracellular transporters and zinc buffering proteins such as metallothioneins (MTs) influence the transport and release of zinc in the blood stream. However, various agents can decrease zinc absorption [98]. For example, it is not uncommon for women of childbearing age to consume calcium supplements for the prevention of osteoporosis or drink water naturally high in calcium. Similar to copper which has an antagonistic relationship with zinc [99–102], calcium might interfere with the absorption of zinc, though this effect is not as well established [103, 104]. Research has shown decreased zinc absorption when different forms of calcium have been ingested following consumption of a zinc dose, suggesting an antagonist relationship between the minerals [105]. Ingestion of high concentrations of iron might also affect zinc uptake [106–108]. Additionally, folic acid is a nutrient commonly prescribed during pregnancy and supplied at higher levels in prenatal supplements. Folic acid has been shown to increase fecal zinc losses, indicating decreased zinc absorption [109–111]. Other dietary constituents that influence zinc availability include phytate and high fructose corn syrup (HFCS). Inositol hexaphosphates and pentaphosphates, the phytate forms that bind to zinc and reduce its availability, are present in staple foods such as wheat, corn, and rice [112, 113]. HFCS is commonly used in the US to sweeten food and drinks with estimated yearly per capita consumption in the US being 12.3 kg in 2012 [114, 115]. Consumption of alcohol leads to a reduced placental zinc transport and it was hypothesized that the consequences of “fetal alcohol syndrome” may unfold not only through the effects of ethanol but also through zinc deficiency [116]. Some drugs are also known to interact with zinc. For example, ACE inhibitors used to treat high blood pressure may decrease blood zinc levels similar to thiazide diuretics, the anticonvulsant valproic acid (VPA) that was already reported to increase the risk for autism upon prenatal exposure [83], tetracycline antibiotics, corticosteroids, acid blockers such as histamine-2 receptor antagonists (H2-blockers), and many neuropsychiatric drugs such as Fluoxetine (Prozac), Paroxetine (Paxil), Sertraline (Zoloft), Citalopram (Celexa), and Venlafaxine (Effexor) [117].

Once absorbed, zinc passes into portal blood and is transported bound to proteins [118]. Placental transport of zinc is a fast process and influenced by the number and size of fetuses present. However, due to low zinc diets or compromised absorption due to increased intake of dietary constituents that reduce the availability of zinc, a zinc deficiency of the embryo may occur. Zinc deficiency might influence embryonic and fetal development through several mechanisms including abnormal nucleic acid metabolism, reduced protein metabolism, reduced rates of tubulin polymerization, high rates of cellular oxidative damage, higher rates of apoptosis, impaired cell migration, and reduced binding of transcription factors and hormones that, among others, affect lymphocytes [119]. These factors will very likely affect GI development.

At least 50 intestinal epithelial differentiation genes have been implicated in development and differentiation of the intestinal epithelium [38], and many of them have a direct relationship with zinc. Among them, adenomatous polyposis coli (APC) is a crucial determinant of cell fate in the murine intestinal epithelium. Loss of APC perturbs differentiation along the enterocyte, goblet, and enteroendocrine lineages and promotes commitment to the Paneth cell lineage through *β*-catenin/Tcf4-mediated transcriptional control of specific markers of Paneth cells, the cryptdin/defensin genes. Conditional deletion promotes Paneth cell differentiation at the expense of enterocyte, goblet, and enteroendocrine cell differentiation [120]. Zinc stabilizes APC levels and induces cell cycle arrest in colon cancer cells [121].

Furthermore, PR domain zinc finger protein 1 also known as BLIMP-1 has an effect on postnatal epithelial maturation, mediating the transition of neonatal intestinal epithelium to adult intestinal epithelium [43]. Caudal-related homeobox (Cdx) regulates intestinal development, differentiation, and maintenance. Cdx1 is required for the transcriptional induction of PPAR*γ* in intestinal cell differentiation [122]. Both variants, Cdx1 and Cdx2, contain a zinc finger motif at their N-terminus.

Gata is another family of zinc finger transcription factors thought to regulate genes involved in embryogenesis. Gata4, -5, and -6 are expressed in various mesoderm and endoderm derived tissues such as heart, liver, lung, gonad, and gut where they play critical roles in regulating tissue-specific gene expression. Gata4, -5, and -6 have been implicated in the regulation of epithelial cell differentiation [123, 124].

Indian hedgehog (IHH) is expressed by mature colonocytes and regulates their differentiation* in vitro* and* in vivo.* IHH binds zinc ions stabilizing the protein and mediating protein-protein interactions [125, 126]. Similarly, Kruppel-like factor 4 (KLF4, formerly GKLF) is a zinc finger transcription factor expressed in the epithelia of the GI tract and several other organs.* In vitro* and* in vivo* studies have suggested that KLF4 plays an important role in cell proliferation and/or colonic epithelial cell differentiation [127].

Matrix metalloproteinases (MMPs) are a family of zinc binding extracellular matrix degrading enzymes. MMP-9 is a zinc dependent endopeptidase, synthesized and secreted in monomeric form as zymogen and contributes to gut microbe homeostasis [128, 129]. Furthermore, MYC-associated zinc finger (MAZ) protein has been implicated as a critical target of the canonical Wnt pathway, which is essential for formation and maintenance of the intestinal mucosa [130, 131].

The Notch signaling pathway promotes proliferative signaling during neurogenesis and is activated in the progenitor domain of the gastrointestinal epithelium influencing binary fate decisions of cells that must choose between the secretory and absorptive lineages in the gut [132]. Notch signaling targets four different receptors referred to as Notch1-4. An important relationship between zinc and the Notch1 signaling pathway can be found. Zinc inhibits Notch signaling by modulating the binding between Notch1 and RBP-Jk [133].

It is thus likely that insufficient zinc supply will affect development of the fetal GI tract contributing to many of the reported GI problems associated with ASD such as metallothionein dysfunction, plasma Cu/Zn inversion, heavy metal overload [4, 83, 134–136],* Candida* and* Clostridium* overgrowth, constipation and/or diarrhea [51, 53], leaky gut, food sensitivities and allergies, inefficient processing of gluten and * *casein [54, 55, 65, 137–139], enzyme deficiency [51, 140–142], vitamin and mineral malabsorption [51, 143–145], inefficient fat digestion and metabolism [146], and esophagitis and GI ulcers [142].

These GI symptoms can give rise to behavioral difficulties, ranging from inattentive or irritable behaviors to self-injury [59]. Several human disorders with GI problems like including inflammatory bowel disease (including Crohn's Disease), irritable bowel syndrome, and obesity have a modulatory influence on social, emotional, and anxiety-like behaviors. Changes in behavior thereby might be based on both acute alterations in brain function as well as alterations during brain development [147–149]. For example, vagal afferent signaling has been implicated modulating mood and affect, including distinct forms of anxiety and fear [150]. Moreover, although the GI symptoms might be transient, long lasting behavioral changes have been reported. In rats, neonatal gastric irritation leads to increase in depression- and anxiety-like behaviors, increased expression of CRF in the hypothalamus, and an increased sensitivity of HPA axis to stress in adults [151]. Thus, it is possible that shared comorbidities such as increased anxiety in ADHD, mood disorders, and ASD correlate with abnormal GI development caused by zinc deficiency or other factors.

The presented model does not exclude the possibility that the GI symptoms are the consequence of altered brain to gut signaling or the consequence of altered gut regulation by the enteric nervous system, which might occur in parallel. Synaptic genes affecting excitatory and inhibitory neurotransmission might lead to alterations in neural or endocrine elements of the enteric nervous system. However, given that a central pathway at synapses related to ASD, Neurexin-Neuroligin-Shank signaling has also been shown to depend in part on the availability of zinc [136], a link between zinc deficiency and brain to gut signaling cannot be excluded.

### 5.2. Prevention and Treatment Strategies

**Figure 4:**
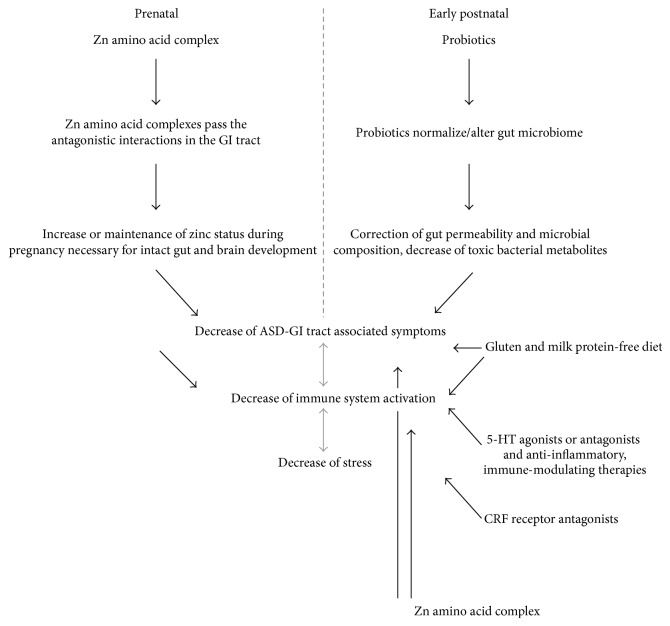
Prevention and treatment strategies. Zinc amino acid complexes might be an effective source to overcome the negative effects of dietary constituents and nutrients in prenatal supplements and help women to maintain adequate zinc status (prenatal prevention, left panel). Zinc supplementation might also be useful in young children with ASD helping to overcome some impairments associated with acute zinc deficiency (diarrhea, impaired immune function, and neurosensory deficits) (postnatal treatment, right panel). Furthermore, young children with ASD might benefit from probiotic therapy that may correct gut permeability, alter microbial composition, reduce burden of bacterial waste products and metabolites, and thereby ameliorate ASD symptoms. Additionally, a gluten and milk protein-free diet was proposed to potentially be beneficial for individuals with ASD. 5-HT signaling may mediate both innate and adaptive responses in the immune system and 5-HT signaling important in the brain and in the GI tract; 5-HT receptors are expressed. Thus, 5-HT3 antagonists or 5-HT4 agonists may have a modulatory effect. Moreover, therapeutics used to treat inflammatory events caused by abnormal GI function (anti-inflammatory and immune-modulating therapies) might be beneficial. Stress is linked to abnormalities in the GI tract and mediated by, among others, the corticotropin-releasing factor (CRF) system on molecular level. The use of CRF receptor antagonists might therefore provide new treatment approaches.

Supplementing women of childbearing age with an effective source of zinc might help mitigate the negative effects of dietary constituents and nutrients in prenatal supplements on zinc availability, helping women attain and maintain adequate zinc status. Zinc amino acid complexes might be advantageous to zinc oxide and zinc sulfate based on better absorption. A combination of the inorganic and amino acid complexed zinc might also be advantageous due to different absorption pathways. Additionally, research has shown that zinc antagonists such as phytate and fiber reduced the bioavailability of zinc from zinc sulfate more than that from a zinc amino acid complex [152]. Provided the amino acid remains complexed to the zinc, interaction of the mineral with dietary components such as phytate and fiber preabsorption can be minimized and zinc can be absorbed into the enterocyte via amino acid transporters versus metal transporters, reducing competition for absorption between zinc in the zinc amino acid complex and other dietary metals [153] (Figure 4).

Although measures to prevent maternal zinc deficiency would be most desired, further treatment strategies emerge from this concept for young children with ASD. For example, the use of probiotics in ASD has been suggested [60, 154, 155]. However, probiotics have been used with variable efficacy and data on the effectiveness of probiotics is currently just emerging with more studies and meta-analyses needed in future. Intriguingly, treatment of the offspring of maternal immune activation (MIA) mice that are known to display features of ASD with the human commensal* Bacteroides fragilis* corrected gut permeability, altered microbial composition, and ameliorated defects in communication, stereotypic- as well as anxiety-like and sensorimotor behaviors [65, 156] (Figure 4). Additionally, a gluten and milk protein-free diet (exclusion of the protein compound gluten found in wheat products and casein contained in dairy) was reported to potentially be beneficial to improve some behaviors in individuals with ASD and reduce intestinal permeability [157, 158]. Elimination of cow's milk protein from the diet of ASD children via restrictive diet improved autistic behavior, while the oral challenge with milk protein seemed to have an opposite effect. When evaluating IgA, IgG, and IgM specific antibodies, autistic children had significantly higher serum levels of IgA antibodies, high levels of IgM antibodies specific for lactalbumin, and IgG and IgM levels for casein [159, 160]. However in a small sample size study with 15 autistic children investigating the effects of a gluten-casein free diet, no significant differences between individuals on the restrictive diet and nontreated controls could be found although some of the parents claimed to have noticed an improvement regarding the child's language, the occurrence of tantrums, and the level of hyperactivity [161]. Thus, although some studies show inflammation of the gut or a leaky gut in ASD and some studies report that gluten and casein showed beneficial effects, given that other scientific publications did not indicate significant improvements, more research is needed to make a recommendation.

In general, the gut microbiome might have great impact on brain development early in life. In an altered microbiome, bacterial metabolites such as 4-ethylphenylsulphate (4EPS) or the neurotransmitter *γ*-aminobutyric acid (GABA) produced from intestinal bacteria might affect brain development and, ultimately, behavior later in life.

Serotonin (5-HT) signaling is not only important in the brain, but also in the GI tract. The 5-HT(1A) receptor plays an important role in the developing brain but is additionally expressed in the gut [162]. 5-HT is released from gut enterochromaffin cells and might contribute to 5-HT signaling in the brain [163]. However, the gut and the brain are not the only sites of action for 5-HT. Its receptors are also present in the immune system where 5-HT signaling may mediate both innate and adaptive responses [164]. It remains to be established whether 5-HT3 antagonists (e.g., Ramosetron) or 5-HT4 agonists can have a modulatory effect in ASD.

Moreover, the intestinal tract has a very important immune function [165]. Besides markers for inflammation, enhanced levels of cytokines and chemokines have been detected in the brain and in the cerebrospinal fluid of children with autism [166, 167]. Therefore, therapeutics used to treat inflammatory events caused by abnormal GI function, such as in inflammatory bowel disease (anti-inflammatory, immune-modulating, and microbiome-modulating therapies) [168], might be a potential source for novel treatment strategies.

Furthermore, stress was implicated in many neuropsychiatric disorders. In particular, prenatal stresses, such as depressive illness, anxiety disorders, and posttraumatic stress disorders, are a risk factor for ASD [83, 169]. Chronic stress may result in GI disorders and immune dysfunction, among others. Maternal stress is able to alter microbial populations and their transmission to the offspring. Thus, stress is also connected to abnormalities in the GI tract, zinc signaling, and the immune system [170, 171]. Many studies support the influence of the corticotropin-releasing factor (CRF) system in stress response. The use of CRF receptor antagonists suggested a significant effect against stress-related behavior, but also hyperalgesia, colonic secretion, and motility [172, 173]. Thus, medications acting on CRF1 and CRF2 receptors that are involved in neuroendocrine, autonomic, behavioral, and visceral responses to stress, such as NBI 27914 and Astressin-2B, respectively, might provide new treatment approaches.

Finally, zinc supplementation might also be useful in young children with ASD. Given that younger individuals with ASD have an especially high risk of zinc deficiency, zinc supplementation will help to overcome some impairments associated with acute zinc deficiency. For example, diarrhea has been linked to zinc deficiency [174, 175] and zinc supplementation was reported to significantly reduce the symptom [176] as well as increase immune function [1, 177] and ameliorate neurosensory deficits associated with zinc deficiency [178] (Figure 4).

Given that ASD is a heterogeneous group of disorders and zinc deficiency or increased intestinal permeability only present in a subset of patients, unless clinical trials use patient populations that are enriched based on this particular clinical history, clinical benefits of any possible treatment will be hard to demonstrate. Additionally, ASD is a neurodevelopmental disorder. Thus, the pathomechanisms already act* in utero*, leading to alternative modeling of the brain. If therapies are to prevent or correct such changes, they may have to be implemented in the perinatal period and may be ineffective in an individual with ASD later in life.

Taken together, we conclude that due to multifaceted effect of zinc on gut development and morphology improving zinc status of the pregnant mother as well as the offspring has the potential to improve gut development of the neonate and potentially mitigate dysfunctions associated with ASD.
